# Short Term Motor-Skill Acquisition Improves with Size of Self-Controlled Virtual Hands

**DOI:** 10.1371/journal.pone.0168520

**Published:** 2017-01-05

**Authors:** Ori Ossmy, Roy Mukamel

**Affiliations:** Sagol School of Neuroscience and School of Psychological Sciences, Tel-Aviv University, Tel-Aviv, Israel; University of Bologna, ITALY

## Abstract

Visual feedback in general, and from the body in particular, is known to influence the performance of motor skills in humans. However, it is unclear how the acquisition of motor skills depends on specific visual feedback parameters such as the size of performing effector. Here, 21 healthy subjects physically trained to perform sequences of finger movements with their right hand. Through the use of 3D Virtual Reality devices, visual feedback during training consisted of virtual hands presented on the screen, tracking subject’s hand movements in real time. Importantly, the setup allowed us to manipulate the size of the displayed virtual hands across experimental conditions. We found that performance gains increase with the size of virtual hands. In contrast, when subjects trained by mere observation (i.e., in the absence of physical movement), manipulating the size of the virtual hand did not significantly affect subsequent performance gains. These results demonstrate that when it comes to short-term motor skill learning, the size of visual feedback matters. Furthermore, these results suggest that highest performance gains in individual subjects are achieved when the size of the virtual hand matches their real hand size. These results may have implications for optimizing motor training schemes.

## Introduction

Vision is an important source of information that plays a significant role in action performance and learning [1, 2]. Indeed, it has been shown that performance on various reaching tasks is lower in the absence of visual feedback [3–5]. Furthermore, visual input has been shown not only to facilitate but also to interfere with simultaneous action performance depending on the congruency level between the two [6–8]. In the context of learning a motor task, visual input is sufficient to introduce significant performance gains even in the absence of overt movement (i.e. training by observation; [9–11]).

When addressing the role of vision, it is important to distinguish between visual input and visual feedback. While the former is independent of the subject’s behavior, the latter is a direct consequence of the subject’s actions. Visual feedback can take two forms: embodied or augmented. Embodied (also termed intrinsic or internal) feedback is related to afferent signals originating from peripheral sensors. Augmented feedback on the other hand relates to feedback from an external source which is based on self-generated actions (e.g. the time on the clock following a 100 meter dash). For a review of the role of augmented feedback in various modalities see [12].

An important dimension of visual feedback originating from the body is effector size. Manipulation of viewed effector size has been shown to have an impact in various domains including pain perception, tactile discrimination, sense of embodiment, and reaching/grasping tasks. For example, two-point tactile discrimination is better in the presence of visual feedback of the hand (relative to tactile stimulation in darkness). Interestingly, tactile discrimination further increases when visual feedback of the hand is magnified [13]. Using the rubber hand illusion, embodied effects have been reported for veridical and enlarged avatar hand size but not for reduced avatar hand size [14]. Additionally, using different rubber hand sizes has been shown to modulate the haptic perception of object sizes [15]. When using whole body illusions, manipulation of perceived body size affects perceived size and distance estimates of objects [16].

In the context of pain perception, visual feedback from the body, and manipulation of its size has also been shown to play a significant role. Visual feedback of the hand while receiving a thermal stimulus has an analgesic effect (relative to no visual feedback) [17, 18], and manipulating the size of the hand’s visual feedback has been shown to influence perceptual and physiological measures of pain in patients [19, 20] and healthy subjects [21, 22].

Finally, it has been demonstrated that humans use continuous visual feedback to correct their concurrent finger movements towards a sequence of visually cued targets [23, 24], and that manipulating the size of visual hand feedback induces changes in mean grip aperture in reaching and grasping tasks [25–27]. Taken together, there is ample evidence supporting the view that the size of visual feedback from the body plays a significant role in various domains including action production. Nonetheless, the role of visual feedback size in short-term motor-skill learning has not been examined. Given the effects mentioned above, it is plausible that manipulation of hand size visual feedback during motor skill training will strengthen visuo-motor coupling which is necessary for learning, and manifest as differences in post-training performance gains.

To examine this issue, we used specialized virtual reality (VR) devices that allowed us to manipulate the visual size of virtual hands controlled by the subject. Size of virtual hands during training on a finger sequence task was manipulated across experimental conditions and corresponding performance gains were evaluated. In addition, the effect of virtual hand size was evaluated in the context of learning by observation (i.e. passive training in the absence of physical movement).

## Materials and Methods

### Subjects

Forty four healthy subjects (29 females, mean age: 26.1, range: 19–27 years) participated in this study after providing written informed consent, and were compensated for their participation either by course credit or money (40 NIS per hr). Two subjects who did not perform the finger movement task correctly during the pre-training or training stages were excluded from the analysis. The Subjects were healthy, right handed, with normal vision and no reported cognitive deficits or neurological problems. Subjects were naïve to the purpose of the study. The experiment was conducted in accordance with the protocol approved by the Tel-Aviv University Ethics Committee.

### Stimuli and Task

**Fig 1:**
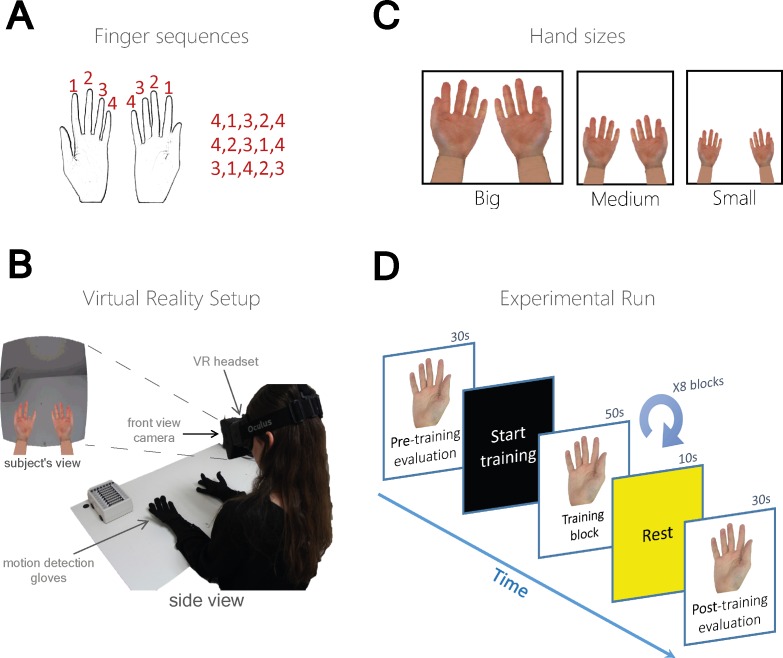
Experimental design and setup. **(a)** Sequence of finger movements to be learned by the subjects. **(b)** Subjects wore a headset and motion sensitive gloves and received visual feedback of virtual hands. The VR devices allowed visual manipulation of online feedback. A camera mounted on the headset allowed embedding the virtual hands and subject’s view inside a natural environment. **(c)** Subjects viewed the embedded virtual hands in three different sizes: big, medium and small (see Materials and Methods, and S1 and S2 Movies). **(d)** Schematic illustration of one experimental session. After instructions, subjects performed the sequence as accurately and rapidly as possible using their right hand (RH) for initial evaluation of performance. Next, subjects were trained according to group–execution with feedback (EXE+OBS) or mere observation (OBS). Finally, they repeated the evaluation test. Each subject underwent a total of 3 different training sessions corresponding to the three different hand sizes. Order of training sessions was counter-balanced across subjects within each group.

Subjects completed three experimental sessions (3 consecutive runs) in which they learned to perform unique sequences of finger movements. Fingers were numbered from index (1) to little finger (4) and subjects were asked to learn a different sequence in each session (3 different sequences: 4-1-3-2-4, 4-2-3-1-4, 3-1-4-2-3; see Fig 1A). Subjects performed the finger sequence task sitting in a chair with their hands forward and palms facing up. Subjects could not see their real hands. Visual feedback of virtual hands was provided through a VR headset used for 3D gaming (Oculus VR, Oculus Rift; see Fig 1B). Subjects wore motion-sensing MR-compatible gloves (5DT Data Glove Ultra 14 sensors) that allow online monitoring of individual finger flexure in each hand. We also used a head-mounted specialized 3D camera (PLAYSTATION Eye digital camera device) to provide online visual feedback of the surrounding environment. The virtual hands were embedded in a specific location in space and were presented only when subjects looked down towards their real hands (See S1 Movie). Delay in feedback is inherently introduced by the sampling rate of both the motion-sensing gloves and the screen. We verified that the software itself does not introduce any additional delays in response. Thus delays between movement time and updating the hands’ animation on the screen was less than 30 ms). Subjects were split into two equal groups (21 subjects per group): physical training group (EXE+OBS), and training by observation group (OBS). Subjects in the physical training group (EXE+OBS) trained on the sequence with their right hand repeatedly in a self-paced manner, while receiving congruent online visual feedback of right virtual hand movement. Subjects in the observation group (OBS) trained by passively observing the virtual right hand performing the sequence, while both their real hands were immobile. Between the groups, subjects were paired such that the pace of virtual hand finger movement in the OBS group was set based on the pace of a corresponding subject from the EXE+OBS group. Within each group, the size of the virtual hands during training varied across three different conditions (See Fig 1C)–big, medium and small. The ratio of the big and small hands relative to medium was 1.44 and 0.6 respectively (see S2 Movie).

### Experimental Design

In the beginning of each session (Fig 1D), subjects were presented with an instructions slide that depicted an illustration of two hands with numbered fingers and a 5 number sequence underneath, representing the sequence of finger movements to be learned. The instructions slide (12 seconds) was followed by a pre-training evaluation stage in which baseline performance level was assessed. Performance level was calculated as the number of times within a fixed time window that the subject performed a complete 5-digit sequence with no errors. During the evaluation, subjects performed the required sequence with the right hand repeatedly as fast and as accurate as possible for 30 seconds (similar to what we used in [11]). At this stage online visual feedback consisted of a display of two virtual hands (medium size) whose finger movements were yoked in real-time to the subjects’ actual finger movements. This stage was identical for both groups. In the ensuing training stage, subjects trained according to their assigned group (OBS or OBS+EXE), under one of the 3 training conditions (big, medium and small). The visual feedback during training consisted of both hands in the size corresponding to the experimental condition. Each training block lasted 50 seconds and was followed by 10 seconds of a yellow blank screen to serve as cue for resting period. The training stage consisted of 8 such training blocks. During training, subjects in the OBS+EXE group were instructed to perform the sequence of finger movements in a self-paced manner, while the subjects in the OBS group were instructed to attend the visual display to learn the sequence. After the training stage, subjects’ performance level was re-evaluated as in the pre-training evaluation stage, with a display of two virtual hands in medium size and finger movements yoked to the actual finger movements. Each subject performed 3 such training sessions (one for each hand size) and each training session was associated with a unique sequence of finger movements to be learned. Order of hand size sessions was counter-balanced across subjects and the finger sequence to be learned was randomized.

### Performance Evaluation

In all evaluation stages, we calculated subject’s performance (P) by counting the number of correctly performed complete 5-digit sequences within 30 seconds. Subject’s performance gain (G) following training was calculated using the formula below:

$$G=\frac{p_{post\_training}-p_{pre\_training}}{p_{post\_training}+p_{pre\_training}}$$ (1)

Where P_post_training_/P_pre_training_ corresponds to the subject’s performance in the post/pre training evaluation stage. Therefore, a positive G index reflects improvement in performance. We calculated the right hand performance gain index for each subject, and each hand size—allowing us to compare differences in improvement under different virtual hand sizes in each subject and across groups.

The motion sensitive gloves allowed us to detect finger movement. This was used for yoking virtual hand movement with real hand movement in the EXE+OBS group, and also for verifying lack of finger movement in the OBS group. Additionally, data from the gloves was used to compare the total amount of self-paced movements performed during the 8 training blocks of the physical training across the different conditions. Each sensor of the glove provided the angle of each finger joint (sampling rate = 16ms) and the subjects always started the training sessions with the hand in the same orientation.

## Results

Across eight training blocks, subjects performed (or merely observed) an average of 141±9.6 full sequence movements during each training condition (averaged across subjects and hand sizes). The number of finger sequences executed (or observed) was not significantly different across the different hand size sessions (minimal p = 0.34 across subjects; rmANOVA). This is not surprising given the fact that subjects were instructed to perform the sequence at their own pace (see Methods).

**Fig 2:**
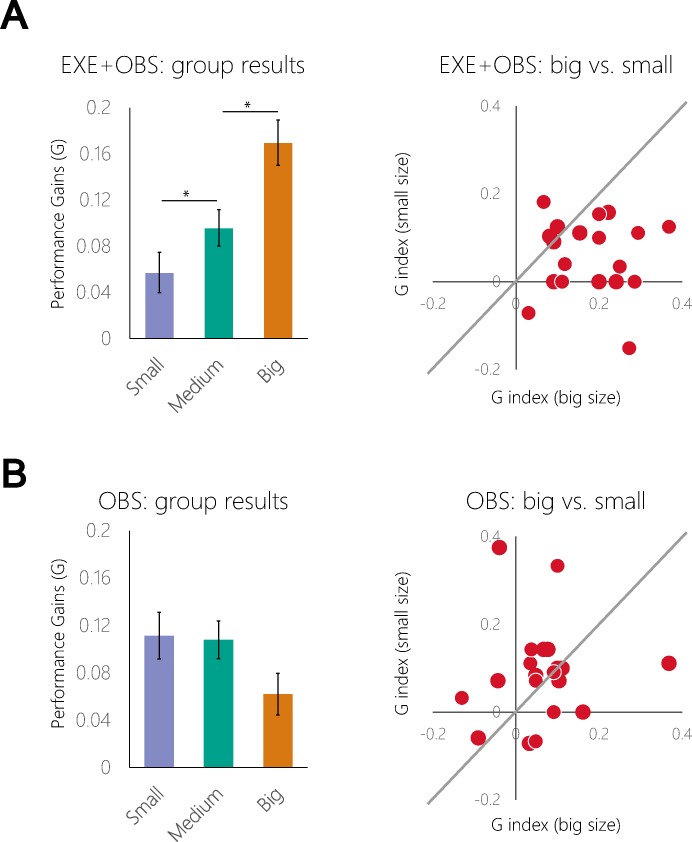
Performance gains and absolute hand size. **(a)** Left panel: group performance gains after training by execution with visual feedback (EXE+OBS group) in three different hand sizes. Performance gains increased with size of virtual hand feedback (asterisks denote significance difference); Right panel: scatter of individual subjects’ performance gains in the big vs. small hand size conditions. The plot demonstrates increased performance gains after training with big hand size vs. training with small hand size (dashed line represents equal performance gains). **(b)** Same as (a) for the observation (OBS) training group. In this group there was no significant difference between the three hand size conditions.

Performance gains (calculated as the accuracy index G) were significantly greater than zero in both groups and all training conditions–demonstrating significant learning following physical execution and also mere observation (maximal p across groups and conditions = 9.8·10^−3^; two-tailed unequal variance t-test). We examined how the size of the virtual hand feedback during training affected subjects’ learning. We found a main effect of hand size in the physical training group (EXE+OBS) (F_2,40_ = 8.28, p<0.01; rmANOVA; See Fig 2A and Table 1). Performance gains after training with big virtual hands was significantly higher than following training with medium virtual hands (p<0.01; post hoc Tukey test). Additionally, training with medium virtual hands resulted in higher performance gains than following training with small virtual hands (p<0.05; post hoc Tukey test). This demonstrates that performance gains following physical training increase with the size of virtual hand feedback. Conversely, in the training by observation group (OBS group) the main effect of hand size was not significant (F_2,40_ = 0.86, p = 0.42; See Fig 2B and Table 2), and subjects did not exhibit a significant difference in performance gains across conditions. We verified that subjects in the OBS group were immobile during the training stages by examining the data from the motion-detection gloves (see Materials and Methods). The maximal angle of each finger during training was not significantly different from the maximal angle during the intervening rest periods. This was true for all fingers in all subjects (minimal p = 0.89; two-tailed paired t-test) supporting the view that indeed the fingers of the subjects in the OBS group during training were immobile. Overall, these results suggest that the visual size of the hand influences subsequent performance gains when it is presented in the context of visual feedback, but not in the context of visual input that is not controlled by the subject.

**Table 1:** EXE+OBS group–performance gains. Individual subject’s performance (P) during pre- and post-training evaluation stages. Each cell represents the number of correctly performed complete 5-digit sequences within 30 seconds. S–subject number.

#	Big size				Medium size				Small size			
	Pretraining	Posttraining	Gindex	Post-pre/pre	Pretraining	Post training	G index	Post-pre/pre	Pre training	Post training	G index	Post-pre/pre
*1*	7	11	0.22	0.57	5	8	0.23	0.6	8	11	0.15	0.37
*2*	16	17	0.03	0.06	13	16	0.1	0.23	15	13	-0.07	-0.13
*3*	9	15	0.25	0.66	12	14	0.07	0.16	14	15	0.03	0.07
*4*	15	18	0.09	0.2	15	16	0.03	0.06	15	18	0.09	0.2
*5*	5	9	0.28	0.8	9	11	0.1	0.22	11	11	0	0
*6*	17	20	0.08	0.17	13	16	0.1	0.23	13	16	0.1	0.23
*7*	14	16	0.06	0.14	14	17	0.096	0.21	9	13	0.18	0.44
*8*	6	9	0.2	0.5	10	11	0.04	0.1	10	10	0	0
*9*	15	19	0.11	0.26	15	20	0.14	0.33	12	13	0.04	0.08
*10*	9	11	0.1	0.22	10	14	0.16	0.4	7	9	0.12	0.28
*11*	16	28	0.27	0.75	14	18	0.12	0.28	19	14	-0.15	-0.26
*12*	11	15	0.15	0.36	10	16	0.23	0.6	12	15	0.11	0.25
*13*	12	18	0.2	0.5	15	16	0.03	0.06	9	11	0.1	0.22
*14*	14	17	0.09	0.21	14	18	0.12	0.28	16	16	0	0
*15*	15	18	0.09	0.2	14	12	-0.07	-0.14	11	11	0	0
*16*	6	13	0.36	1.16	10	10	0	0	7	9	0.12	0.28
*17*	6	11	0.29	0.83	9	11	0.1	0.22	8	10	0.11	0.25
*18*	8	12	0.2	0.5	11	13	0.08	0.18	11	15	0.15	0.36
*19*	11	18	0.24	0.63	6	8	0.14	0.33	14	14	0	0
*20*	15	18	0.09	0.2	14	19	0.15	0.35	15	18	0.09	0.2
*21*	16	20	0.11	0.25	18	18	0	0	17	17	0	0

**Table 2:** OBS group–performance gains. Same as Table 1 for the OBS group.

#	Big size				Medium size				Small size			
	Pre training	Post training	G index	Post-pre/Pre	Pre training	Post training	G index	Post-pre/pre	Pre training	Post training	G index	Post-pre/pre
*1*	14	15	0.03	0.07	9	13	0.18	0.44	12	15	0.11	0.25
*2*	9	11	0.1	0.22	6	5	-0.09	-0.16	4	8	0.33	1
*3*	13	12	-0.04	-0.07	9	15	0.25	0.66	5	11	0.37	1.2
*4*	15	16	0.03	0.06	15	16	0.03	0.06	15	13	-0.07	-0.13
*5*	12	15	0.11	0.25	14	16	0.06	0.14	9	11	0.1	0.22
*6*	12	11	-0.04	-0.08	14	15	0.03	0.07	13	15	0.07	0.15
*7*	13	10	-0.13	0–0.3	13	12	-0.04	-0.07	15	16	0.03	0.06
*8*	12	10	-0.09	-0.16	5	12	0.41	1.4	9	8	-0.05	-0.11
*9*	10	11	0.04	0.1	10	11	0.04	0.1	11	13	0.08	0.18
*10*	13	16	0.1	0.23	5	13	0.44	1.6	13	15	0.07	0.15
*11*	13	14	0.03	0.07	12	14	0.07	0.16	9	12	0.14	0.33
*12*	9	11	0.1	0.22	9	11	0.1	0.22	9	11	0.1	0.22
*13*	10	11	0.04	0.1	13	16	0.1	0.23	13	15	0.07	0.15
*14*	10	12	0.09	0.2	12	15	0.11	0.25	10	12	0.09	0.2
*15*	13	18	0.16	0.38	14	19	0.15	0.35	13	13	0	0
*16*	10	12	0.09	0.2	11	12	0.04	0.09	2	7	0.55	2.5
*17*	10	11	0.04	0.1	11	11	0	0	8	7	-0.06	-0.12
*18*	7	8	0.06	0.14	5	7	0.16	0.4	6	8	0.14	0.33
*19*	6	13	0.36	1.16	14	16	0.06	0.14	12	15	0.11	0.25
*20*	5	6	0.09	0.2	12	13	0.04	0.08	8	8	0	0
*21*	6	7	0.07	0.16	7	8	0.06	0.14	6	8	0.14	0.33

**Fig 3:**
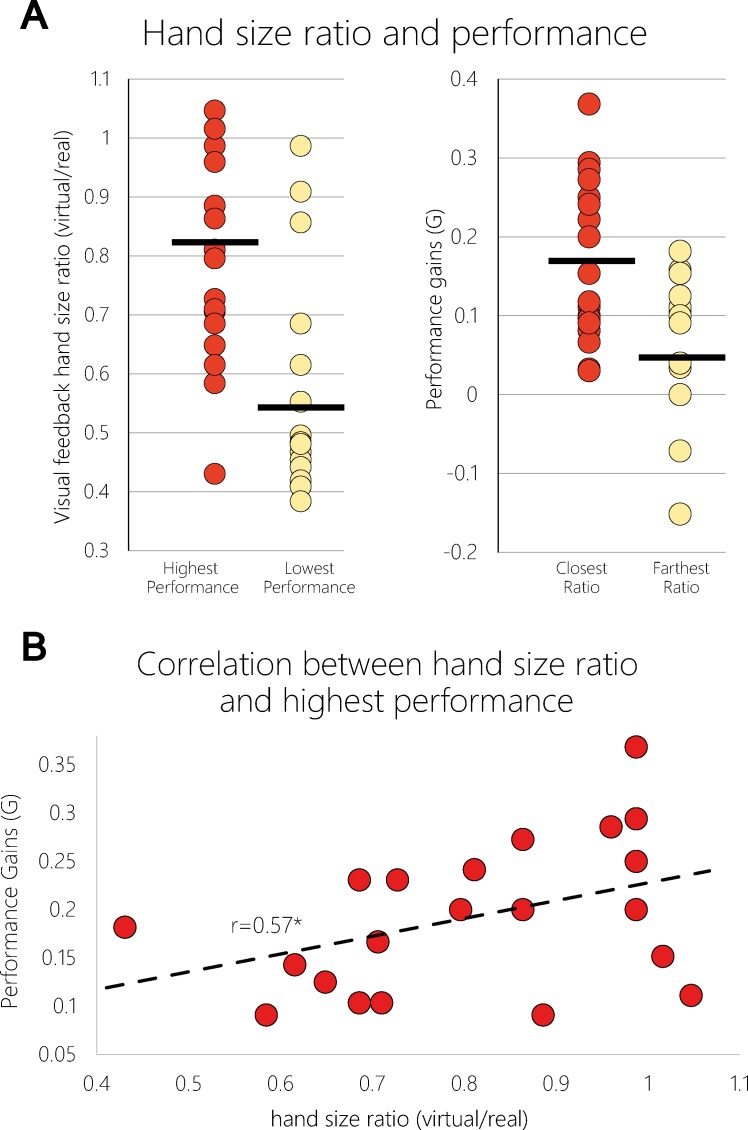
Performance gains and relative hand size. **(a)** Left panel—for each subject in the EXE+OBS group, we calculated the ratio between virtual and real hand size in the condition with the highest performance gains and the condition with the lowest performance gains separately. Each dot represents a single subject and the dark lines represent the group mean. There is a significant difference between the hand size ratios of the high and low performance groups (mean hand size ratio high = 0.81; low = 0.55; p<0.01; two-tailed paired t-test). Right panel–for each subject we plot the performance gains in the condition in which the ratio (virtual/real) was closest to 1 (red; mean = 0.16) and the condition in which the ratio was farthest away from 1 (white; mean = 0.05). Performance gains in the two conditions were significantly different (p<0.01; two-tailed paired t-test). **(b)** Scatter plot showing individual hand size ratio (virtual/real) against performance gains in the condition with highest performance gains of each subject. The correlation (dashed line) was significant (p<0.01; Pearson correlation).

So far, our analysis demonstrates that at the group level, absolute visual size of virtual hand feedback influences subsequent performance gains in the physical training condition. Next, we examined how the relative difference between the actual size of the subject’s hand, and the size of the virtual hand presented on the screen relates to modulations in performance gains of individual subjects. The actual hand size of the subjects in the EXE+OBS group was 18.14cm on average (range 14–21.7) and 18.02cm (range 15.5–21) in the OBS group (no significant difference between the groups: p = 0.81; two tailed equal variance t-test). Hand size was measured as the distance in centimeters from the tip of the middle finger to the wrist. To examine the relationship between relative hand size and performance gains, for each subject in the OBS+EXE group we calculated the ratio between the size of the virtual hand they observed and their real hand (a ratio of 1 indicates perfect match in size). Actual hand size ratios across subjects ranged from 0.37 to 1.23. For each subject we chose the training conditions (big, medium or small) that yielded the highest and the condition that yielded the lowest performance gains. Fig 3A (left panel) presents the distribution of hand size ratios for all subjects in the conditions resulting in the highest (red) and lowest (white) performance gains. As can be seen, the highest performance gains across the EXE+OBS subjects were obtained following training with higher hand-size ratios (mean hand size ratios 0.81 and 0.55 respectively; p<0.01; two-tailed paired t-test).

We also compared performance gains for each subject in the condition in which hand size ratio was closest vs. the condition in which it was farthest from 1. To this end, for each subject we chose the condition in which the hand-size ratio was closest or farthest from 1 and plotted corresponding performance gain (Fig 3A right panel). We found significantly higher performance gains in conditions in which the hand size ratio was closer to 1 (p<0.01; two-tailed paired t-test). The correlation between hand size ratio (virtual/real) of individual subjects and performance gains in the condition with highest performance gains was significant (r = 0.57, p<0.01; Pearson Correlation; See Fig 3B). We also examined whether actual hand size of the subjects was related to performance gains. To this end, subjects were split into two equal groups (median split) according to the size of their real hands. The difference in performance gains between these groups was not significant for any of the 3 hand sizes and also for the averaged performance gain across hand size conditions (p = 0.14; two-tailed t-test). Taken together, these results imply that higher compatibility between actual and observed hand size feedback (rather than actual hand size per se) is associated with increased motor learning.

## Discussion

Visual information is a fundamental factor in motor behavior, and many studies have considered the effects of visual information on motor skill acquisition [8, 12, 28–30], even without concurrent physical movement [9, 11]. The size of visual feedback from the body has been previously examined in various domains including pain perception [17, 18, 20], tactile discrimination [13], sense of embodiment [14, 15], and reaching/grasping tasks [26, 31] but not with respect to short term motor skill learning. Here we used VR devices to manipulate the size of virtual hands controlled by the subject, embedded in the real environment as captured by a camera. These devices, unlike the use of minifier or magnifier glasses, enabled us to specifically re-scale the hands, without scaling the environment (an effect that also changes the perception of depth and distance). We show that the size of visual feedback of the hand during motor training has a significant effect on subsequent performance gains. Performance gains increased with the size of the virtual hands but only in the context of visual feedback, when the subjects actively controlled the virtual hand and not in the context of visual input (as in training by observation). Previous studies in motor control showed that participants changed their hand kinematics following changes in hand-size visual feedback [26, 27]. To the best of our knowledge, the current study is the first to demonstrate that rescaling the visual feedback during training can boost the motor performance in humans.

It could be argued that larger hand sizes are more visually salient than smaller hand sizes, and therefore induce higher performance gains due to increased attention. However, under such circumstances, one would expect similar increases in performance gains in the OBS group which we did not find. Alternatively, it could be argued that the subjects in the OBS group did not show a similar increase in performance gains due to lack of attention. However, since subjects in this group showed significant increases in performance gains for all hand sizes, this alternative explanation seems less likely to underlie the lack of sensitivity to hand size in this group. Taken together, a simple differences in salience/attention across conditions or groups does not provide a satisfactory explanation for the current pattern of results.

It has been argued that training by action observation engages similar learning processes as physical training [32, 33]. A large body of evidence using neuroimaging in humans supports this notion by showing overlapping patterns of cortical activity during action observation and action execution (for meta-analysis see [34]). Here, indeed both training by mere observation and physical training with visual feedback resulted in significant performance gains. However, we found that visual parameters like the size of the observed hand have a different behavioral effect on the two types of trainings suggesting separate underlying mechanisms. A follow-up neuroimaging study exploiting the protocol presented here will be needed to explain this discrepancy at the neural level.

Evaluation of performance in both groups was conducted with a medium size visual feedback irrespective of the visual hand size used in the training stage. Thus compatibility of the hand size during the training stage and pre/post training evaluation stages was highest in the medium condition and lowest in the big/small conditions. Such differences in size compatibility of visual information between the training and evaluation steps could in principle introduce an advantage favoring the performance evaluation in the medium size condition. Nonetheless, we did not find performance gains in the medium hand size condition to be highest in either training group. Furthermore, since both big and small hands are incompatible to a similar extent, one would expect similar performance gains in the two conditions. However, this does not correspond with our finding of increased performance gains from small to large hand size in the OBS+EXE group.

Our results indicate that in the context of feedback, performance gains increased with a larger size of the virtual hand. Furthermore, our results indicate that performance gains increase with the compatibility between the size of the virtual hand and the size of the subject’s real hand (ratio close to 1). Since most of the hand size ratios in our study were less than 1, it is difficult from the current data to unequivocally conclude that increased performance gains are due to greater absolute size of visual hand display, or rather due to greater compatibility between the virtual and actual hand size. Within our experimental display setup (the VR 3D goggles), we were limited in the range of virtual hand sizes we could display. This limitation prevented us from using very large hand sizes (importantly ratios greater than 1). Therefore we cannot disambiguate whether absolute size or relative size of virtual hand is the key factor for optimal increases in performance gains and this remains an open question for future research. Finally, our results are limited to short-term (within-session) learning effects therefore further research is needed to examine the role of hand-size feedback across multiple sessions.

The effect of hand size in the current study was specific to the feedback condition, in which the subjects controlled the visual display of virtual hand movement. In contrast, no size effect was obtained in the observation group. Thus, a neural-based explanation for our findings should account for the integration among visual and tactile senses [35]. A possible mechanism which deserves inspection relies on neurophysiological and behavioral evidence suggesting that during tool use, there is an expansion of visuotactile receptive fields in parietal and pre-motor regions to the spatial limits of the tool (for review see [36, 37]. Enlarged visual feedback of the hands also results in transient plastic enlargement of visuo-tactile receptive fields in such regions [38, 39] to encompass the enlarged hands and nearby peri-personal space [40, 41]. Such enlargement of receptive fields could facilitate visuomotor coupling which plays an important role in motor skill learning [42, 43]. The current findings thus bear relevance for the learning process in children in which the ratio between the observed “template” and the actual effector size is greater than 1 as typically happens when children imitate adults [44, 45].

By examining the effects of hand size on short-term motor learning, we show that larger hand sizes (and increased compatibility with actual hand size) in the context of visual feedback result in increased performance gains. This sets the stage for future neurophysiological studies examining the motor-perception mechanisms underlying motor learning in humans. Additionally, our results have key consequences on exploration of optimal learning protocols in real-word situations such as motor-skill acquisition in children, and patient rehabilitation.

## Supporting Information

S1 Movie Illustration of experiment setup. (MP4)

S2 Movie Illustration of experiment stimuli. (MP4)
